# Supplementary material for: Determinants of institutional delivery service utilization in Nepal
Source: PLoS One. 2023 Sep 21;18(9):e0292054. doi: 10.1371/journal.pone.0292054 (PMC10513198; doi:10.1371/journal.pone.0292054)
Supplement: Attachment — Submitted filename: review.docx [file pone.0292054.s001.docx]

Can be accepted.
