# Supplementary material for: Determinants of institutional delivery service utilization in Nepal
Source: PLoS One. 2023 Sep 21;18(9):e0292054. doi: 10.1371/journal.pone.0292054 (PMC10513198; doi:10.1371/journal.pone.0292054)
Supplement: Attachment — Submitted filename: Response to reviewer.docx [file pone.0292054.s002.docx]

Dear editor and reviewers, thank you very much for your suggestions. We have extensively changed the abstract, and main text of the manuscripts as per your suggestions and feedbacks.

Editor: Please also include the roles and academic qualification of each author. I also suggest to have more review of relevant literature in the field.

Authors: Thank you very much for your suggestion. We have written the roles and academic qualification of each author while submitting in the submission system. As per your suggestion, we have added more review of the relevant literature in the field, in the manuscript.

**Abstract section:**
Editor: The abstract should be rewritten. It must contain the status of global status of ID, why it is important to research, and why it is important in Nepal’s context. Also, required to brief about the methodology in the abstract section. The use of data source methods, statistical test has been not properly mentioned in the section.

Authors: Thank you very much for your suggestion. We have changed and added lines in the background and methods section of abstract as per your suggestion.

Editor: In the conclusion section, you wrote about the role of local level. Have you really relied on the findings of the study for this recommendation?

Authors: Thank you very much for your suggestion. We have removed the role of local level from the conclusion section of the abstract and retain the part which relies on the findings of our study.

**Background:**
Editor: Need to have an additional literature review on the subject matter.
What is the SDG target for MMR and how is the progress towards SDG? What are the unfinished agenda of MDGs in Nepal related to maternal and neonatal health?
You mentioned about only a handful of the studies used national level survey data to assess the factors for institutional delivery. Why? What is the research gap on the subject matters?

Authors: Thank you very much for your suggestion. We have addressed all your suggestion in the background section of the manuscript in the from line numbers 55 to 85.

**Methods:**
Editor: Include the information, how the research was carried out? Secondary or primary data analysis? How the approval was taken from MICS program to use the data for further analysis?

Authors: Thank you very much for your suggestion. We have written “The present study was based on data from the Nepal Multiple Indicator Cluster Survey (MICS) 2019” to indicate the data being used in the study as secondary data. Since, we did not participate in the design of the study and data was download from the MICS site, we have written “The detailed methodology of the survey can be accessed from the Nepal MICS 2019 report. The website of the MICS program makes the anonymized survey dataset freely available under request in line numbers 104 to 106 with references. The approval was taken from MICS program to use the data for further analysis via requesting them in an email.

Editor: Conceptual framework and expert opinion: what are the theories that you used in the present research to formulate the conceptual framework?

Authors: Thank you very much for your suggestion. Conceptual framework was solely based on the literature review and expert opinion. We have clearly mentioned this in line number 120.

**Results:**
Editor: The whole result section should be rewritten and should present in an analytical way. Only the describing the table won’t be enough. Please review other scientific literature for describing the results.

Authors: Thank you very much for your suggestion. We have reviewed other scientific literatures published in PLOS journal and others as well. We have rewritten some sentences of the result section as per your suggestions.

Editor: Have you ever tried to explore the husband role’s for institutional delivery in Nepal? If not, please use all available variable for analysis.

Authors: Thank you very much for your suggestion. Yes, we tried to explore the husband role in institutional delivery in our study however we could not do it because there is no variable in women data set which could assess the husband role in institutional delivery. Moreover, the man data set which provides variable related to men could not be merged into women data set as there is no key variable given to link these two data sets.

**Conclusions and recommendations**
Mostly described findings only. Why only the government role’s been discussed, what about the roles of other stakeholder’s role in increasing institutional delivery in Nepal.

Authors: Thank you very much for your suggestion. We have also added the term “stakeholder’s effort” in line number 370 of the conclusion and recommendations section of the manuscript.

**Reviewer #1:Review Comments**

1. What are the gains in the reduction of maternal mortality in Nepal and nation specific strategies since 2015?

Authors: Thank you so much for you suggestion. We have added the gains in reduction of maternal mortality and nation specific strategies since 2015 in line number 62-74.

2. The objective is not SMART.

Authors: Thank you so much for you suggestion. We have adjusted the objective to make it SMART.

3. Why systematic sampling while selecting the final HHs?

Authors: The present study was based on data from the Nepal Multiple Indicator Cluster Survey (MICS) 2019 conducted by the Central Bureau of Statistics in collaboration with the United Nations Children’s Fund. We did not participate in the design of the study and data was download from the MICS site. In the Multiple Indicator Cluster Survey (MICS), systematic sampling is often used in the second stage of sampling for selecting households within the selected clusters. This method is employed for practical reasons and to ensure an efficient and representative sample.

4. Why don’t you assessed for the socio-economic and cultural factors affecting the institutional delivery in Nepal. What are the exact sources of the conceptual framework?

Authors: Thank you very much for the question. We have incorporated important socio-economic and cultural factor available in the data sets that affects the institutional delivery in Nepal. We have included religion, ethnicity, wealth status, household head’s sex, age and education, and women’s age and education in our model to determine the potential socio-economic and cultural factors associated with institutional delivery. The conceptual framework was based on the literature review and expert opinion.

5. Who collected the data? How was the data collected? What are the measures taken to ensure data quality assurance?

Authors: Nepal Multiple Indicator Cluster Survey Nepal 2019 team who worked under Central Bureau of Statistics collected the data with the help of trained survey enumerators. They visit selected households and administer structured questionnaires through face-to-face interviews.

MICS utilizes a multipronged approach to data‐quality assurance. Interviewers are extensively trained for an average of three to four weeks prior to conducting fieldwork, with only those mastering the tools being selected. Fieldworkers are organized into teams, with a supervisor who coordinates field activities and revisits households for quality control. During fieldwork, data are transmitted and stored to a cloud server on a daily basis. Every week, survey managers run field‐check tables that measure data‐quality indicators, disaggregated by team and interviewer. This allows corrective action to be taken while interviewers are in the field.

6. Why “The first model consisted 137 of external environment factors. Furthermore, household contextual factors were added in the 138 second model, while individual factors were added in the third model.”?

Authors: Thank you very much for the question. The model consisted of external environment factors in first model and household contextual factors were added in the second model while individual factors were added in the third model. External environment factors may have a contextual influence on individual outcomes. By including these factors first, we accounted for the contextual variation in the data and estimated the contextual effects on the outcome variables. This helped to capture the impact of the broader environment on individuals within the model.

7. How was the data merged? Did both the data set and source have similar variable? If there was difference, how did you manage it? Is there data that is discarded?

Authors: Thank you very much for the question. The UNICEF MICS website provides information to merge the data set.

Source:<https://mics.unicef.org/files?job=W1siZiIsIjIwMTkvMDQvMDEvMTQvMDAvMjQvNzM0L0ZBUV9NZXJnaW5nX01JQ1NfZGF0YV9maWxlc18yMDE5MDIyOC5kb2N4Il1d&sha=c3117037366d1001>

We merged two data sets namely women and household data set. When merging women’s data files with their households, we used the cluster numbers (variable HH1) and household numbers (variable HH2) as key variables available in the both datasets.

After merging two different data sets i.e., mother and household, a total of 14,805 observations were found. 1,950 observations of married women aged 15-49 years with a live birth in the last two years by place of delivery of the most recent live birth were selected and retrieved. After removing five observations with missing values: the place of delivery (n=1), health insurance (n=3) and household head education (n=1), a total of 1,945 observations remained. Finally, 13 observations which did not fit the criteria for the place of delivery were removed and 1,932 observations were considered in the final analysis.

8. Language, grammar and sentences were not written as per the standard of the scientific paper. E.g., A sentence beginning with numeric.

Authors: Thank you very much for your suggestion. We have checked the language, grammar and sentences again.

9. The result should be simple, logical and coherent.

Authors: Thank you very much for your suggestion. Some of the sentences from the result section have been rewritten in order to make them more simple, logical and coherent.

10. The discussion and the conclusion should be in line with the objective of the study.

Authors: Thank you very much for your suggestion. We have tried to adjust the paragraphs in the discussion and conclusion.

**Reviewer #2:** A well written manuscript. However, it will be better if the title is rewritten in the statement instead of the question in alignment with the general objective. Otherwise, it is okay.
can be accepted.

Authors: Thank you so much for your suggestion. We have changed the title of the manuscript as “Determinants of Institutional Delivery Service Utilization in Nepal” in statement form in alignment with the objective of the study.
