# Supplementary material for: Determinants of institutional delivery service utilization in Nepal
Source: PLoS One. 2023 Sep 21;18(9):e0292054. doi: 10.1371/journal.pone.0292054 (PMC10513198; doi:10.1371/journal.pone.0292054)
Supplement: Attachment — Submitted filename: Response to reviewer.docx [file pone.0292054.s003.docx]

Additional Editor Comments:

Editor: Dear Authors, I suggest you to rework, correct or answer on the following aspects: -
Line 122. Please take care of it.

Authors: We have deleted it.

Editor: Line 143: Can you please make clear about the line. After the approval of whom, where and how?

Authors: Thank you so much for the comment. The first author requested access for the MICS Nepal 2019 datasets to the UNICEF MICS Team through their website. Subsequently, the MICS Team granted access to the data via email. Following data access approval, the first author proceeded to download the dataset directly from the MICS website's survey page. We have added few words to make it clearer in in the analysis subsection of the manuscript.

Editor: Ethical consideration section- you mentioned about ethical consideration and approval from Central Bureau of Statistics. Are they the legal body to provide ethical clearance for your study.

Authors: We have not taken ethical clearance from Central Bureau of Statistics for our study. The survey protocol for MICS was approved by the Central Bureau of Statistics as per the Statistical Act (1958) in September 2018. The Statistical Act enables Central Bureau of Statistics to carry out surveys according to the government’s ethics protocol without involving an institutional review board. Our study was based on data from the MICS 2019 conducted by the Central Bureau of Statistics.

Editor: You have mentioned about two heading - Statistical Methods AND data management. Is there any opportunity to make these or three into one. OR is it really required?

Authors: Thank you for the suggestion. We have merged this two sub-heading to one, making it data management and analysis sub-section.

Editor: While reading, I did not see when the study was conducted? Please mention it.

Authors: Thank you for the suggestion. Nepal MICS 2019 was conducted by the Central Bureau of Statistics from May to November 2019 as a part of sixth-round of the global MICS household program, with the technical and financial support from United Nations Children’s Fund Nepal. We have utilized the data obtained from this study. We have added this information in the data source subsection to make it clearer.

Editor: If you are referring some critical appraisal checklist for your study for self-evaluation, it can help you to better improve your study. Therefore, I may suggest you to once refer to such critical appraisal checklist for your study.

Authors: Thank you so much for suggestion. We have used STROBE Statement to self-evaluate our study. We have added this to the strength and limitation section of our study.

Reviewer #3: It seems like that authors have addressed the raised comments by the editor and the other reviewers, otherwise well formulated peace of work. To be a final manuscript i suggest to write more explanation about husbands’ role although you mentioned some in the review answer but add little more in the actual text, as this is important perspective. Tables need extra explanation and arrangement.

Authors: Thank you so much for suggestion. We have added more explanation about husband’s role in the discussion section of our manuscript as per your suggestion. We have added extra explanation for table 3 as per your suggestion.

Reviewer #4: Thank you for submitting your research with us, some references need to be updated (References No. 3,4,5,7,9,29,39,44) and all references need editing to be written in the same style.

Authors: Thank you so much for suggestion. We have updated the references and edited them to be written in the same style.
